# Commentary on ‘The role of coagulopathy and subdural hematoma thickness at admission in predicting the prognoses of patients with severe traumatic brain injury: a multicenter retrospective cohort study from China’

**DOI:** 10.1097/JS9.0000000000001720

**Published:** 2024-05-28

**Authors:** Yiacob T. Kibret, Abass O. Ajayi, Mahendra P. Singh, Mahalaqua N. Khatib, Quazi S. Zahiruddin, Sarvesh Rustagi, Ayush Anand

**Affiliations:** a Myungsung Medical College, Addis Ababa, Ethiopia; b Ahmadu Bello University Teaching Hospital, Zaria, Nigeria; c B.P. Koirala Institute of Health Sciences, Dharan, Nepal; d Medical Laboratories Techniques Department, Al-Mustaqbal University, Hillah, Babil, Iraq; e Division of Evidence Synthesis; f South Asia Infant Feeding Research Network (SAIFRN), Division of Evidence Synthesis, Global Consortium of Public Health and Research, Datta Meghe Institute of Higher Education, Wardha; g School of Applied and Life Sciences, Uttaranchal University, Dehradun, Uttarakhand; h Center for Global Health Research, Saveetha Medical College and Hospital, Saveetha Institute of Medical and Technical Sciences, Saveetha University, Chennai; i MediSurg Research, Darbhanga, India

*Dear Editor,*

Traumatic brain injury (TBI) remains a leading cause of morbidity and mortality worldwide, with an urgent need for reliable prognostic tools to guide clinical decision-making^1–3^. Traditional models often fall short in accurately predicting outcomes due to the complex interplay of various factors involved in brain injury. In the evolving landscape of TBI management, the study by Chen *et al*.^4^ presents a significant advancement in our understanding and ability to predict patient outcomes. This study’s innovative approach of integrating coagulopathy and subdural hematoma (SDH) thickness into a prognostic model offers a more nuanced and potentially more accurate assessment of patient outcomes. The study leverages a robust dataset of 1006 patients over a 6-year period across multiple large medical centers, enhancing the reliability and generalizability of its findings in the Chinese population. The authors employed rigorous statistical methods, including receiver operating characteristic curves, nomograms, calibration curves, and decision curve analysis, to validate their predictive model. The use of machine learning to rank the importance of predictive indicators adds a contemporary and sophisticated dimension to the research, ensuring that the most significant factors are appropriately weighted in the prognostic model.

The integration of coagulopathy and SDH thickness into a single predictive model (X1) represents a significant step forward. Coagulopathy, a common complication in severe TBI, is often associated with poor outcomes due to the complex balance between bleeding and thrombosis^3^. SDH thickness further complicates the clinical picture, often necessitating surgical intervention^3,5^. By combining these two factors, the X1 model provides a more comprehensive and clinically relevant tool for predicting patient outcomes at 6 months post-discharge. The study’s findings suggest that the X1 model offers superior predictive accuracy compared to coagulopathy alone, with higher C-index values in both training and validation cohorts. This improved accuracy could facilitate more informed clinical decisions, potentially leading to better-targeted interventions and resource allocation.

Despite its strengths, the study’s retrospective design limits the ability to establish causality. First, prospective studies are needed to confirm these findings and assess their applicability in real-time clinical settings. Second, the study’s geographic limitation to Chinese medical centers necessitates further validation in diverse populations to ensure broader applicability. Third, the study also opens avenues for exploring the mechanistic underpinnings of the observed associations. Understanding the biological interplay between coagulopathy and SDH thickness could inform the development of targeted therapies aimed at mitigating these complications and improving patient outcomes.

In conclusion, this study represents a noteworthy contribution to the field of TBI research. The novel integration of coagulopathy and SDH thickness into a prognostic model has the potential to enhance clinical practice significantly. As we continue to strive for precision in medical care, such innovative approaches are crucial in addressing the complexities of severe TBI.

## Ethical approval

Ethical approval is not applicable for this correspondence article.

## Consent

Informed consent is not applicable for this correspondence article.

## Source of funding

None.

## Author contribution

Y.T.K.: conceptualization, project administration, supervision, validation, writing – original draft, and writing – review and editing; A.O.A.: supervision, validation, writing – original draft, and writing – review and editing; M.P.S., M.N.K., Q.S.Z., and S.R.: writing – original draft and writing – review and editing; A.A.: supervision, validation, writing – original draft, and writing – review and editing.

## Conflicts of interest disclosure

There are no conflicts of interest.

## Research registration unique identifying number (UIN)

None.

## Guarantor

Abass Oluwaseyi Ajayi.

## Data availability statement

Data sharing is not applicable to this article as no new data were created or analyzed in this study.

## Provenance and peer review

Not commissioned, externally peer-reviewed.

## Assistance with the study

None.
